# Narrow Margins: Aerobic Performance and Temperature Tolerance of Coral Reef Fishes Facing Extreme Thermal Variability

**DOI:** 10.1111/gcb.70100

**Published:** 2025-03-06

**Authors:** Grace O. Vaughan, Daniel M. Ripley, Matthew D. Mitchell, Dain McParland, Jacob L. Johansen, Holly A. Shiels, John A. Burt

**Affiliations:** ^1^ Marine Biology Laboratory New York University Abu Dhabi Abu Dhabi UAE; ^2^ Division of Cardiovascular Science, Faculty of Biology, Medicine and Health The University of Manchester Manchester UK; ^3^ Yas SeaWorld Research and Rescue Abu Dhabi UAE; ^4^ Hawaii Institute of Marine Biology University of Hawaii at Manoa Honolulu Hawaii USA; ^5^ Mubadala Arabian Center for Climate and Environmental Sciences (Mubadala ACCESS) New York University Abu Dhabi Abu Dhabi UAE

**Keywords:** climate change, metabolism, ‘plastic floors’, plasticity, respirometry, thermal tolerance

## Abstract

Climate change is driving rising average sea temperatures and the intensification of thermal variability. Tropical coral reef fishes have evolved under thermally stable conditions to function optimally within a narrow temperature range, with many currently living close to their upper thermal limits. However, recent work has demonstrated that some species possess additional capacity, such as reductions in basal metabolic rates (i.e., ‘plastic floors’), to compensate for the acute effects of thermal challenges when assessed over multigenerational timeframes. In this study, we use the ‘plastic floors and concrete ceilings’ hypothesis to generate and then test predictions regarding the thermal physiology of reef fishes in the world's hottest and most thermally variable coral reef ecosystem (southern Arabian/Persian Gulf). By comparing three species of reef fishes ( *Scolopsis ghanam* , *Ecsenius pulcher* and *Cheilodipterus novemstriatus* ) from the southern Arabian/Persian Gulf, with an annual temperature range of 18.0°C–36.5°C, to conspecifics from nearby but more thermally benign (~21.0°C–32.0°C) reefs in the Gulf of Oman, we find enhanced upper thermal limits and a broadening of the temperature performance curves for aerobic scope in the Arabian/Persian Gulf, but no evidence for changes in basal metabolic rates (‘plastic floors’). Despite these conserved increases in temperature tolerance, the summer thermal safety margins of Arabian/Persian Gulf fishes were 1.47°C lower than those of conspecifics from the Gulf of Oman, demonstrating that while the temperature tolerance of tropical coral reef fishes is somewhat plastic over multigenerational timeframes, its rate of change is likely insufficient to keep pace with the rising average temperatures and growing thermal variability expected under climate change.

## Graphical abstract

Over long timeframes, animals may adapt or acclimate to their environment. By comparing fishes from ‘typical’ and thermally extreme coral reef habitats, we find limited differences in their metabolic physiology and show that those from the extreme environment are living closer to their maximum temperature. This suggests that coral reef fishes may lack the capacity to cope with the rising temperatures and growing thermal variability expected under climate change.

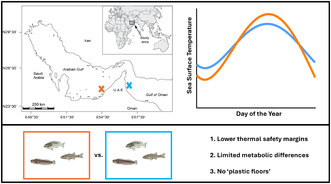

## 1 Introduction

Climate change is driving an increase in both average sea surface temperature and thermal variability (Easterling et al. 2000; Meehl and Tebaldi 2004). Changes in the thermal environment have profound consequences for the ecology of fishes, including shifting species distributions, reducing maximum body sizes and altering the timing of reproductive events (Deutsch et al. 2020; Pauly 2021). Interest in the consequences of changing ocean temperatures has grown substantially in recent years as its effects are becoming more pronounced on fishes of ecological and commercial concern. However, our understanding of the physiological mechanisms driving changes to species ecology and fitness remains unclear, despite being fundamental to predicting population persistence during climate change (Little et al. 2020; Pörtner and Farrell 2008). Furthermore, concomitant with rising average temperatures is a predicted increase in thermal extremes throughout the century (Easterling et al. 2000; Meehl and Tebaldi 2004). While a substantial number of studies have investigated the effects of rising average temperatures on fishes, few integrate these predicted changes in thermal variability (Vázquez et al. 2017).

Metabolic rates are often used in studies of temperature‐driven changes in fishes (Clark et al. 2013; Norin and Clark 2016; Schulte 2015) owing to the theoretical link between aerobic scope and biological fitness, even though a direct mechanistic association between the two is heavily debated (Jutfelt et al. 2018; Lefevre et al. 2021). Acute warming typically increases metabolic demands (Schulte 2015), reducing the aerobic scope available to fuel ecologically important activities. Aerobic scope describes the absolute aerobic surplus above basal maintenance activities that can be used for processes such as digestion, migration and courtship. Thus, aerobic scope is hypothesised to be associated with biological fitness (Pörtner et al. 2017); however, there is limited empirical evidence of this relationship (Lefevre et al. 2021). Over longer timeframes, organisms may compensate for the acute effect of temperature on metabolic rates through physiological acclimation (i.e., active plasticity, Earhart et al. 2022; Johansen et al. 2021; Zambie et al. 2024), which often shifts the metabolic surplus back towards pre‐warming levels (Havird et al. 2020). Evidence is mounting that over longer time scales, other mechanisms such as transgenerational plasticity, genetic and epigenetic adaptation and developmental plasticity may complement physiological acclimation, providing further capacity to withstand warming (Sandblom et al. 2016; Pilakouta et al. 2020; Donelson et al. 2011; Earhart et al. 2022; Ripley et al. 2023; Lock et al. 2024; Pottier et al. 2022; Grenchik et al. 2013).

The importance of long‐term plasticity in fishes is exemplified by the ‘plastic floors and concrete ceilings’ hypothesis, which suggests that basal physiological parameters (‘floors’) show considerable thermal plasticity under chronic warming, while physiological maxima (‘ceilings’) are relatively fixed (Sandblom et al. 2016). For example, reductions in standard metabolic rate are often observed in fishes exposed to chronically warmed conditions, while changes in maximum metabolic rates are not (Sandblom et al. 2016). This ‘plastic floor’ has been observed in several eurythermal species of chronically warmed fish, including perch ( *Perca fluviatilis* , Sandblom et al. 2016) and three‐spined stickleback ( *Gasterosteus aculeatus* , Pilakouta et al. 2020), leading to suggestions that it could represent a universal response to rising average temperatures (Jutfelt 2020). However, climate change is characterised not only by rising temperatures but also by the concomitant intensification of thermal variability, which may introduce additional or conflicting pressures (Scheuffele et al. 2024), resulting in a distinct long‐term response of fish populations (Morash et al. 2018). While the ‘plastic floors and concrete ceilings’ hypothesis has strong support in populations experiencing rising average temperatures, it is important that we assess the breadth of its utility by testing its ability to predict the long‐term physiological response of fish populations to other temperature‐driven stressors. Presently, there is a lack of empirical evidence testing predictions made from the ‘plastic floors and concrete ceilings’ hypothesis in populations that have experienced extreme thermal variability, across multiple seasons and generations, within natural settings.

Mechanisms facilitating plasticity over long‐term timeframes have the potential to be especially important in coral reef fishes, which are thought to lack substantial acclimation capacity over short timeframes (days—weeks) owing to their thermally stable evolutionary histories (Donelson et al. 2011; Rummer et al. 2014; Johansen et al. 2021). Tropical coral reef fishes typically display narrow thermal performance curves (TPCs), with a distinct thermal optimum and steeply declining performance at both sub‐ and supra‐optimal temperatures (Rummer et al. 2014), resulting in small thermal safety margins. Given their lack of short‐term acclimation capacity, determining the potential for tropical reef fishes to use longer‐term mechanisms to cope with rising average temperatures and growing thermal variability is crucial for assessing their survival and effectively managing populations during this era of climate change.

The Arabian/Persian Gulf (AG) is the most thermally extreme coral reef habitat on earth, with an annual temperature range around 20°C and summer maxima exceeding 36°C (Coles 2003; Vaughan et al. 2019). The AG is a young sea, with reef fauna colonising the modern coastline just 6000 years ago (Kennett and Kennett 2006; Smith et al. 2022). Thus, the AG serves as an opportunity to explore how coral reef fishes have naturally coped with extreme temperatures and variability, which are today akin to ‘end of century’ projections for coral reefs globally, over multigenerational time scales. In this study, we compare the performance of three evolutionarily distinct species of tropical reef fishes from the AG to conspecifics from the nearby, but more thermally benign reefs in the Gulf of Oman (GO, ~21°C–32°C). By contrasting three species across two distinct regions, we aim to elucidate (1) whether populations of coral reef fishes experiencing large seasonal temperature fluctuations over multigenerational timeframes show different thermal tolerances and metabolic performance curves compared to those from more thermally benign reefs and (2) whether these changes follow the principles of ‘plastic floors and concrete ceilings’ described in chronically warmed populations. We predict that (1) fishes from the AG will show broader TPCs for aerobic scope than conspecifics from the GO, allowing the maintenance of physiological performance across their full seasonal temperature range; (2) the broader TPC for aerobic scope will be driven by a low thermal sensitivity and overall reductions in standard metabolic rate, but no differences in maximum metabolic rate, adhering to the principle that physiological floors show greater plasticity than ceilings and (3) AG fishes will show enhanced thermal limits, but smaller thermal safety margins during both the summer and winter periods.

## 2 Methods

### 2.1 Species Collection and Maintenance

**FIGURE 1:**
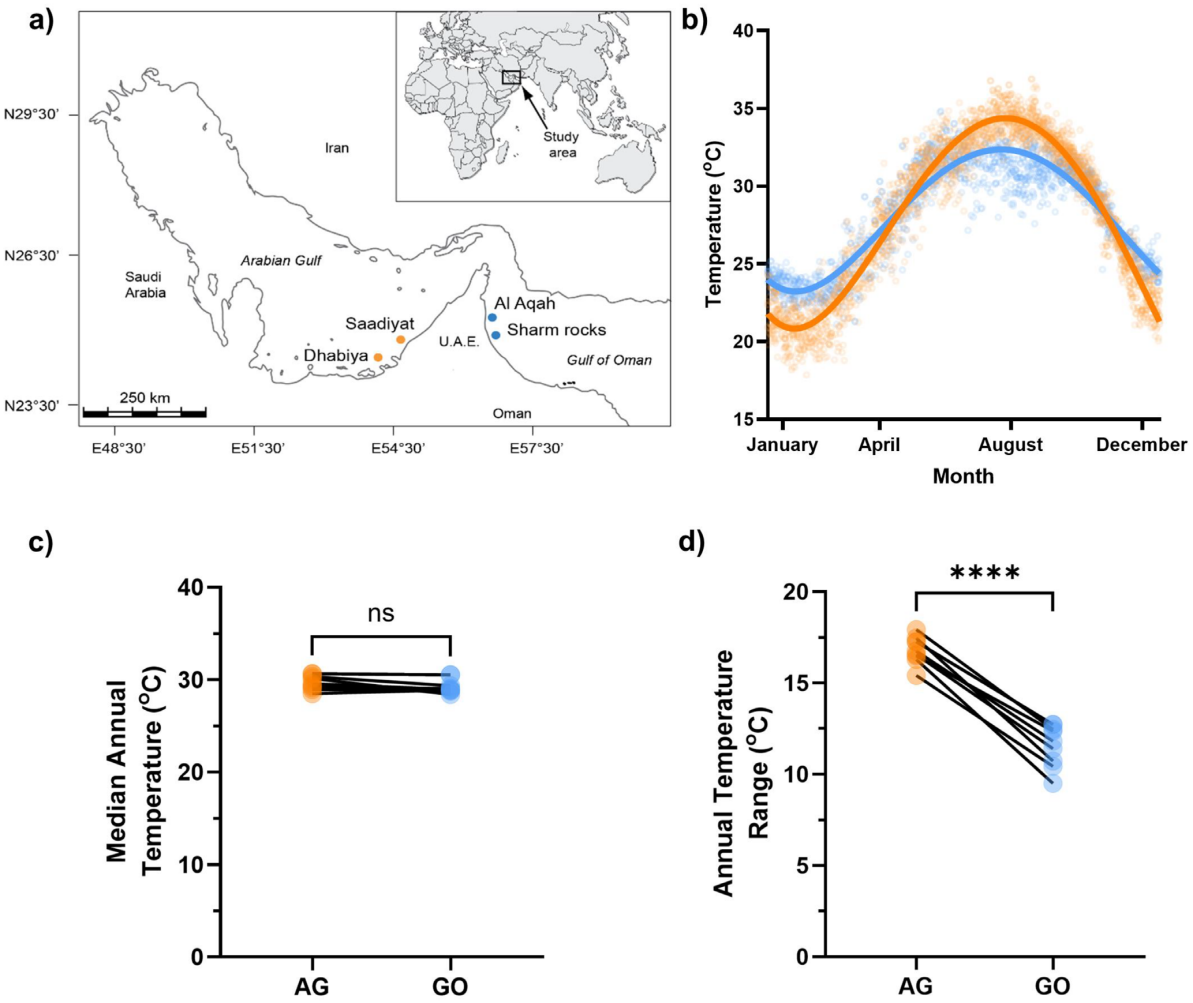
(a) location of the study sites within The Arabian Gulf (orange, Saadiyat and Dhabiya reefs) and the Gulf of Oman (blue, Al Aqah and Sharm Rock reefs). (b) Sea surface temperatures at Dhabiya reef in the Arabian Gulf (orange) and Al Aqah reef in the Gulf of Oman (blue) from January 2010—October 2019. Curves fitted using a fourth‐order polynomial equation (Arabian Gulf, *r* ^2^ = 0.94, Gulf of Oman, *r* ^2^ = 0.87). (c) Annual median and (d) Annual range of sea surface temperatures at Dhabiya and Al Aqah reefs from 2010 to 2018. Asterisks denote statistically significant differences (****p < 0.0001). Sea surface temperature data are downloaded from aqua modis daily sst 4 km L3 (NASA OBPG 2020; Kilpatrick et al. 2015, 2019). Map lines delineate study areas and do not necessarily depict accepted national boundaries.

All procedures were approved by the Institutional Animal Care and Use Committee at New York University Abu Dhabi. *Scolopsis ghanam* (Nemipteridae), *Ecsenius pulcher* (Blenniidae) and *Cheilodipterus novemstriatus* (Apogonidae) were chosen for this study owing to their high local abundance and differing ecological roles, trophic levels and maximum body sizes (Froese 2022; Randall 1995; Vaughan et al. 2021). *Ecsenius pulcher* is a small, cryptobenthic herbivore found primarily hiding in crevices. *Cheilodipterus novemstriatus* is a similarly small benthic omnivore that feeds primarily in the water column, whereas *S. ghanam* is a larger carnivore (Froese 2022; Froese and Pauly 2024; Springer 1988; Russell 1990; Gon 1993). These species are evolutionary distant fishes separated by ~112 million years and represent three distinct families (Kumar et al. 2022). Therefore, these fishes provide the opportunity to evaluate performance metrics both within closely related populations (intra‐specific) as well as across evolutionary traits (interspecies). Fishes were collected ( *S. ghanam* , mass = 5.30–68.30 g; *E. pulcher* , mass = 0.09–3.87 g; *C. novemstriatus* , mass = 0.29–3.37 g) from two reefs in the AG and two reefs in the GO (four total sampling sites, Figure 1a) using barrier nets ( *S. ghanam* ) or hand nets and a mild clove oil anaesthetic ( *E. pulcher* and *C. novemstriatus* ). All reefs have comparable benthic composition, similar depth (6–8 m) and are within commercially unfished coastal waters (Bento et al. 2016).

Experimental temperatures were selected to represent the winter minima, spring average and summer maximum for both the AG (18°C, 22°C, 27°C, 31.5°C and 35.5°C) and the GO (22°C, 27°C and 31.5°C). To ensure natural acclimation to ambient temperatures, fishes were collected when the AG reefs reached five target temperatures (18°C, 22°C, 27°C, 31.5°C and 35.5°C) (±0.5°C), allowing the assessment of traits from naturally acclimatised fishes across their full range of environmental temperatures (Figure 1b). All fishes were held for a minimum of 10 days and until feeding stabilised before the experiments began. As the GO does not reach 18°C or 35.5°C, a subset of fish caught at 22°C and 31.5°C were acclimated in the laboratory to 18°C or 35.5°C, respectively. Temperature ramping prior to acclimation occurred at a rate of 0.5°C per day, and fishes that were laboratory acclimated (GO 18°C and 35.5°C) were held at their final temperature for a minimum of 3 weeks prior to experimentation (Johansen et al. 2024, 2021). While *E. pulcher* from the GO were able to acclimate to 18.0°C and 35.5°C, acclimation was stopped for individuals showing signs of distress, which included all individuals of *S. ghanam* and *C. novemstriatus* from the GO at 35.5°C. As a result, data are available from *S. ghanam* and *C. novemstriatus* from the GO at 18.0°C, but not 35.5°C.

All fish were housed in temperature‐controlled (±0.1°C, Willhi series 1436 controller) 118 L Perspex tanks for the entire study, except when undergoing trials (respirometry or critical thermal limits). Tanks included shelters and were maintained with a supply of recirculated, aerated, filtered sea water (285 L sump) under a 12:12 light: dark cycle. Fish were fed to satiation twice daily with food pellets (New Life Spectrum marine fish formula, 1 mm pellets for *C. novemstriatus* and *E. pulcher* and Aquamax grower 400 starter feed, 2.4 mm pellets for *S. ghanam* ). *E. pulcher* and *C. novemstriatus* were maintained in groups of 10 individuals per tank, whereas, owing to their larger size, *S. ghanam* was held in groups of four individuals per tank. Physiological performance across the seasonal temperature range was examined across five different metrics: critical thermal maxima (CT_max_), critical thermal minima (CT_min_), standard and maximum metabolic rates (SMR and MMR) and aerobic scope (AS). Following trial completion, all fish were humanely euthanised to allow collection of ecologically and physiologically relevant tissues for additional studies unrelated to this publication (including gonads, otoliths, hearts, gills, liver and muscle).

### 2.2 Respirometry

Intermittent flow respirometry was used to measure the oxygen uptake rates (ṀO_2_) of individual fish ( *S. ghanam* mean mass ± SD = 32.45 ± 12.75 g; *E. pulcher* mean mass ± SD = 1.43 ± 0.71 g; *C. novemstriatus* mean mass ± SD = 1.58 ± 0.54 g) at their respective acclimatisation temperatures. For each experiment, up to four fish were run in parallel. Prior to a trial, fish were fasted for 24 h to ensure a post‐absorptive state (Chabot et al. 2016). MMR was achieved by chasing individual fish for 3 min, followed by 1 min of air exposure (Clark et al. 2013). Immediately following the air exposure, fish were placed into custom‐built respirometry chambers (1.2 L for *S. ghanam* and 0.175 L for *E. pulcher* and *C. novemstriatus* ) housed within a water bath supplied with aerated, temperature‐controlled (±0.1°C) water from a sump, and ṀO_2_ was measured. Post‐exercise ṀO_2_ was monitored for 6 min, or until 80% O_2_ saturation was reached, after which time the chamber was flushed with aerated water from the surrounding water bath. Respirometry cycles were 10 min, with flushing and measurement times varying based on water temperature and fish body size, but always ensuring that the O_2_ saturation in the chambers equilibrated with that of the buffer tank. Fish were then left in the chambers for 16–20 h overnight to achieve SMR. To ensure minimal bacterial growth, all water flowed through a filtration system, and the entire respirometry equipment was rinsed with a 10% bleach solution, followed by freshwater, between each trial.

Maximum metabolic rate was taken as the ṀO_2_ during the first measurement period following the exhaustive exercise. Standard metabolic rate was estimated as the lowest 20th percentile of measurements taken throughout the duration of the trial, excluding the first 5 h where ṀO_2_ was elevated following the exhaustive chase (Chabot et al. 2016). Background respiration was estimated in each empty chamber for 30 min prior to, and 30 min immediately following, each trial. To obtain the background corrected SMR and MMR values, the first background measurement was deducted from the MMR measurement, and the post‐trial background measurement was deducted from the SMR measurement. Aerobic scope was calculated as the difference between the background corrected MMR and SMR values. Temperature coefficient (Q10) values, which are a measurement of thermal sensitivity describing the rate at which a process changes with temperature, were then calculated for the metabolic parameters.

### 2.3 Critical Thermal Maximum and Minimum

Fish went through either a CT_max_ or a CT_min_ trial. Those who had previously gone through a respirometry trial had a minimum of 24 h recovery prior to having their thermal tolerance assessed. Where the number of fish in a group was low, we focused on either CT_max_ or CT_min_, based on the acclimatisation temperature (CT_max_ in warm acclimatised fish and CT_min_ in colder acclimatised fish). For each experiment, batches of up to four fish were run simultaneously. Fish were transferred into individual plastic mesh baskets placed within a 50 L aerated water tank maintained at their holding temperature and left to settle for 20 min prior to trial commencement. Water temperature was increased (CT_max_ trials) or decreased (CT_min_ trials) at a rate of 0.1°C min^−1^ (Jobling 1981) using a temperature controller digital thermostat (Willhi WH1436A 10A 110 V) controlling the influx of water from a 60 L water bath connected to a heater/chiller (Hailea HC‐2200BH). The temperature at which an individual displayed loss of equilibrium ( *S. ghanam* and *C. novemstriatus* ), or failure to maintain its position/right itself following a gentle nudge ( *E. pulcher* , a bottom‐dwelling species) was recorded (LOE). The latter approach was required for *E. pulcher* as it sits on the benthos and shows limited visible signs of losing equilibrium. Once the critical thermal limit was observed, the individual was immediately transferred back to its original tank for recovery. Internal body temperature at LOE was calculated following the approach of Schurmann et al. (1991) and used as the critical thermal limit (CT_max_ or CT_min_). This calculated internal temperature was used as body temperature is known to lag behind water temperature in a manner that is dependent upon body mass. Thermal safety margins (TSMs) were then calculated as the difference between an individual's critical thermal limit at their respective summer (AG, 35.5°C; GO, 31.5°C) or winter (AG, 18.0°C; GO, 22.0°C) acclimation temperatures and the average summer maximum or winter minimum temperature recorded at each reef (January 2010–October 2019, NASA OBPG 2020; Kilpatrick et al. 2015; Kilpatrick et al. 2019).

### 2.4 Data Analysis and Statistics

Data analyses were performed in R (http://www.R‐project.org/, R Core Team 2019) and GraphPad Prism 10.3.0. The respirometry and critical thermal limit data in this manuscript are available through Figshare at https://doi.org/10.6084/m9.figshare.28342562.v2 (Vaughan et al. 2025). The sea surface temperature data are available at https://doi.org/10.5067/MODAM‐1D4N9 (NASA OBPG 2020).

All thermal safety margin data were normally distributed (Shapiro–Wilk test, *p* > 0.05) and so were contrasted between the AG and GO regions using *t*‐tests. The only exception was the summer thermal safety margins of *C. novemstriatus* , which were contrasted between regions using a Mann–Whitney *U* test. Annual temperature range displayed a normal distribution (Shapiro–Wilk test, *p* > 0.05) and was compared between sites using a *t*‐test. Median annual sea surface temperature did not conform to a normal distribution (Shapiro–Wilk test, *p* < 0.05) and so was compared between the AG and GO using a Mann–Whitney *U* test. All *p*‐values calculated for *t*‐tests and Mann–Whitney *U* tests were two‐tailed.

Prior to statistical analysis, ṀO_2_ data were body mass corrected following the protocol of Johansen et al. (2024). Mass correction was necessary owing to differences in body size between fishes from the two regions, whereby fishes from the GO are generally larger than conspecifics from the AG (Brandl et al. 2020; D'Agostino et al. 2021). Briefly, each metabolic trait (SMR, MMR and AS) was regressed against body mass, and the slope of each relationship was used to adjust the respective values to the mean body mass of each species. Separate scaling coefficients were calculated for each species and metabolic trait. As thermal limits in nature are absolute values, CT_max_ and CT_min_ were not scaled to body mass to maintain their ecological relevance. Instead, body mass was included as a variable in the models of CT_max_ and CT_min_.

Linear models were created for each species to assess the effects of temperature, region (AG vs. GO), and their interaction on each of the measured physiological traits. Model residuals were examined by visual inspection of the residual plots and by Shapiro–Wilk normality tests. Where a model's residuals did not conform to a normal distribution, response variables were transformed using Tukey's ladder of powers transformation (Tukey 1977), and the model was re‐run. In cases where transformation did not result in normally distributed residuals, analysis was instead performed in a non‐parametric framework using a permutational multivariate analysis of variance (PERMANOVA) in the R package ‘vegan’ (Dixon 2003; Anderson 2014).

Linear models were used to analyse *E. pulcher* AS, SMR, MMR and CT_max_; *S. ghanam* CT_max_ and CT_min_ and *C. novemstriatus* AS, SMR and CT_min_. PERMANOVAs were used to analyse *E. pulcher* CT_min_; *S. ghanam* AS, SMR and MMR and *C. novemstriatus* MMR and CT_max_. Post hoc pairwise comparisons were computed using estimated marginal means (‘emmeans’ package, Lenth 2018) or the ‘pairwise.adonis’ function (Martinez Arbizu 2020) for linear models and PERMANOVAs, respectively. P values were adjusted for multiple comparisons testing using the ‘fdr’ method.

## 3 Results

### 3.1 Thermal Tolerance

**FIGURE 2:**
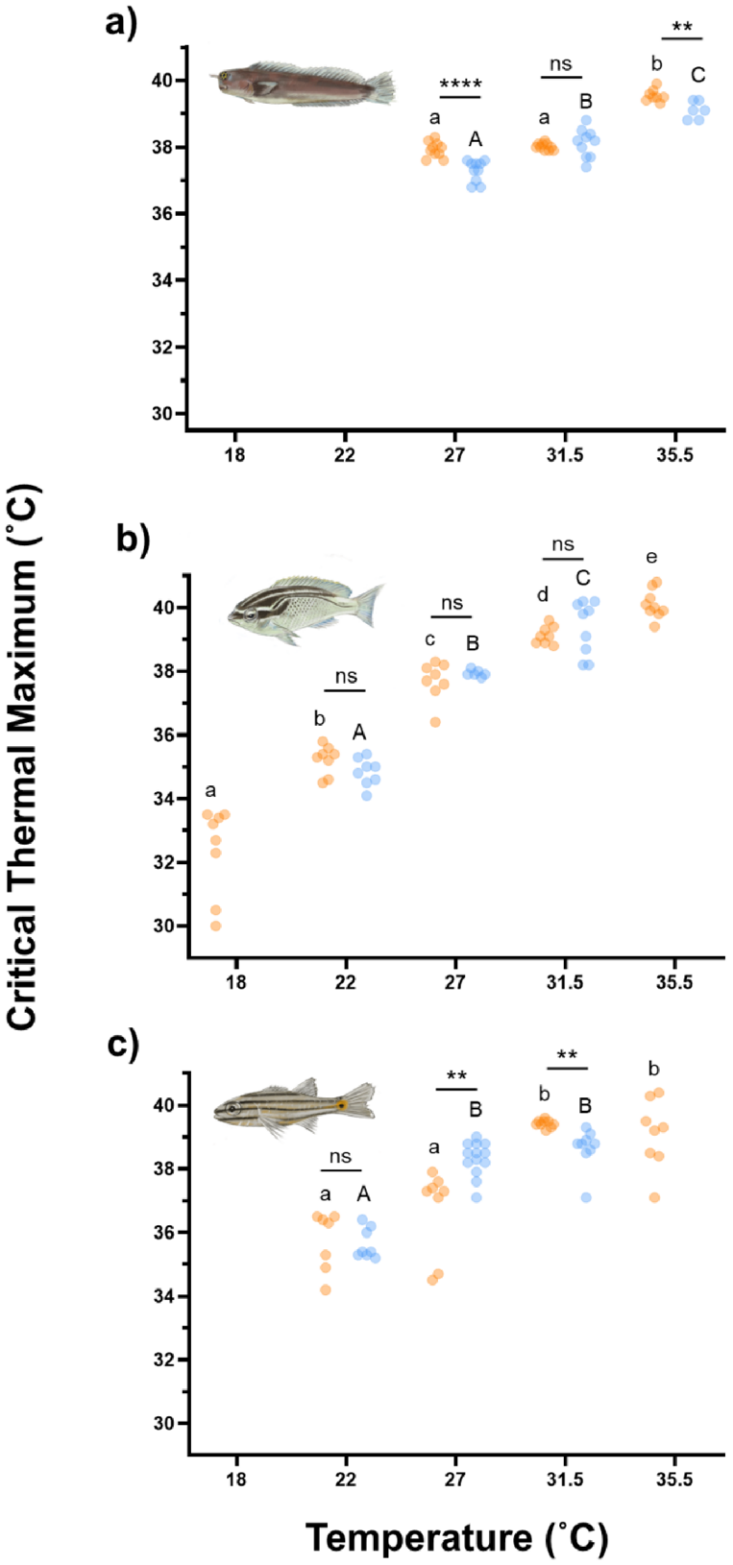
Critical thermal maximum of *Ecsenius pulcher* (a), *Scolopsis ghanam* (b) and *Cheilodipterus novemstriatus* (c) from the Arabian Gulf (orange) and the Gulf of Oman (blue) at five temperatures. Lowercase letters denote significant differences (fdr < 0.05) between temperature treatment groups in fishes from the Arabian Gulf. Uppercase letters denote significant differences (fdr < 0.05) between temperature treatment groups in fishes from the Gulf of Oman. Asterisks denote significant differences (**fdr < 0.01, ****fdr < 0.0001) between fishes from the different regions at a given temperature. *N* = 54, 64 and 62 for *E. pulcher* , *S. ghanam* and *C. novemstriatus* . Where data are present, *n* = 6–13 per species, per region and per temperature.

**FIGURE 3:**
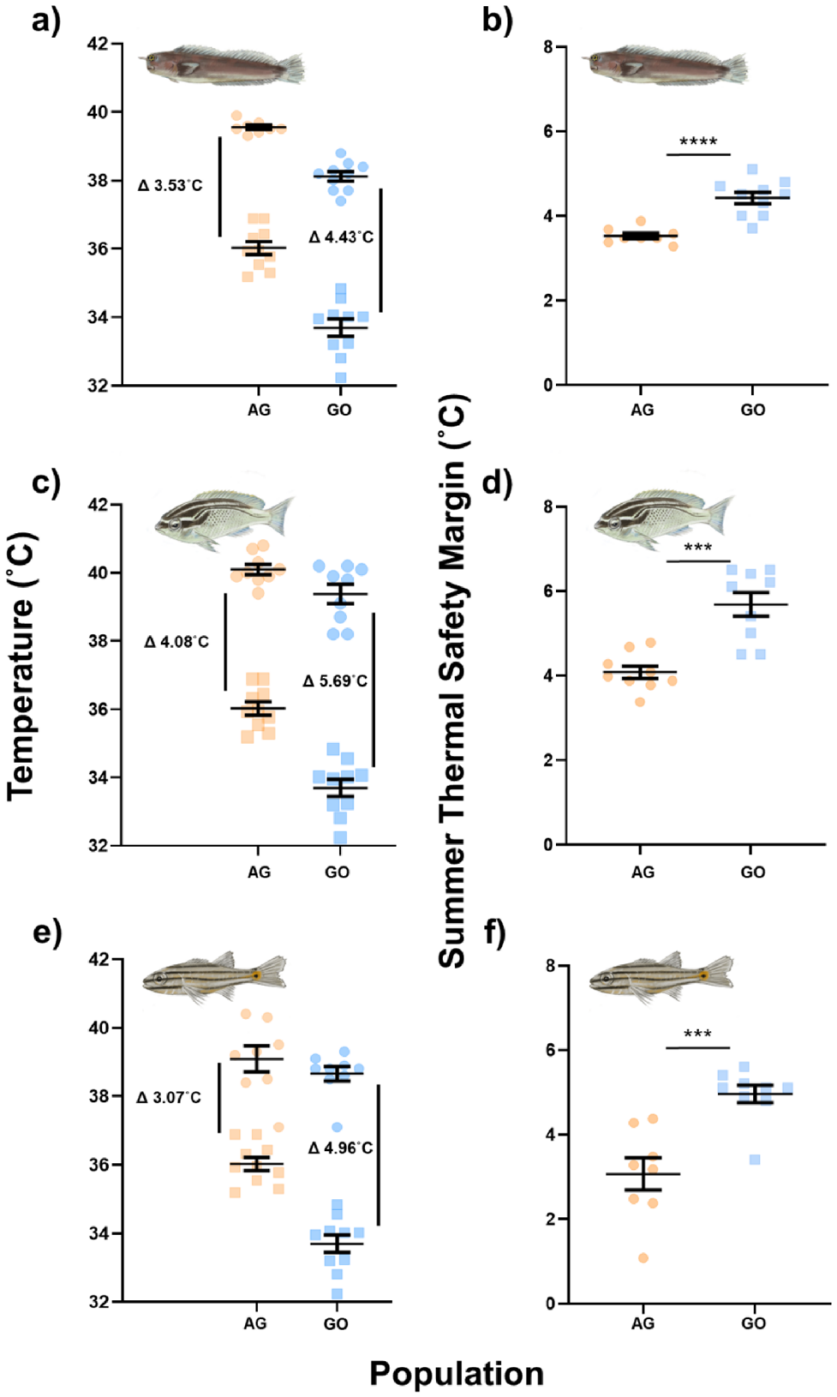
(a, c, e) Critical thermal maximum (circles) and peak annual sea surface temperatures (squares, 2010–2019). (b, d, f) Summer thermal safety margins. Data show *Ecsenius pulcher* (a, b), *Scolopsis ghanam* (c, d) and *Cheilodipterus novemstriatus* (e, f) from the Arabian Gulf (orange) and the Gulf of Oman (blue) naturally acclimatised to their respective summer temperatures (Arabian Gulf, 35.5°C; Gulf of Oman, 31.5°C). Delta represents the mean thermal safety margin (CT_max_—environmental maximum temperature). *n* values: *E. pulcher* , AG = 8, GO = 10; *S. ghanam* , AG = 9, GO = 9; *C. novemstriatus* , AG = 8, GO = 9. Bars and error bars show the mean ± standard error.

In accordance with our prediction, AG fishes showed enhanced upper thermal limits that manifest as either a greater CT_max_ at the highest acclimation temperature (Figure 2a, *E. pulcher* CT_max_ ± SE, AG = 39.55 ± 0.065, GO = 39.10 ± 0.11, *p* = 0.0038) or an ability to acclimate to higher temperatures than GO fishes (35.5°C, *S. ghanam* , *C. novemstriatus* ). Despite their improved thermal limits, all three species of AG fishes displayed significantly lower TSMs at their respective peak‐summer temperatures than conspecifics from the GO (Figure 3, AG = 35.5°C, GO = 31.5°C, *E. pulcher* TSM ± SE, AG = 3.53 ± 0.065, GO = 4.43 ± 0.13, *t* = 5.56, df = 16, *p* < 0.0001; *S. ghanam* TSM ± SE, AG = 4.08 ± 0.15, GO = 5.69 ± 0.28, *t* = 5.08, df = 16, *p* = 0.0001; *C. novemstraitus* TSM ± SE, AG = 3.07 ± 0.38, GO = 4.96 ± 0.21, *p* = 0.0005). On average, TSMs during the summer were 1.47°C lower in AG fishes than those from the GO.

**FIGURE 4:**
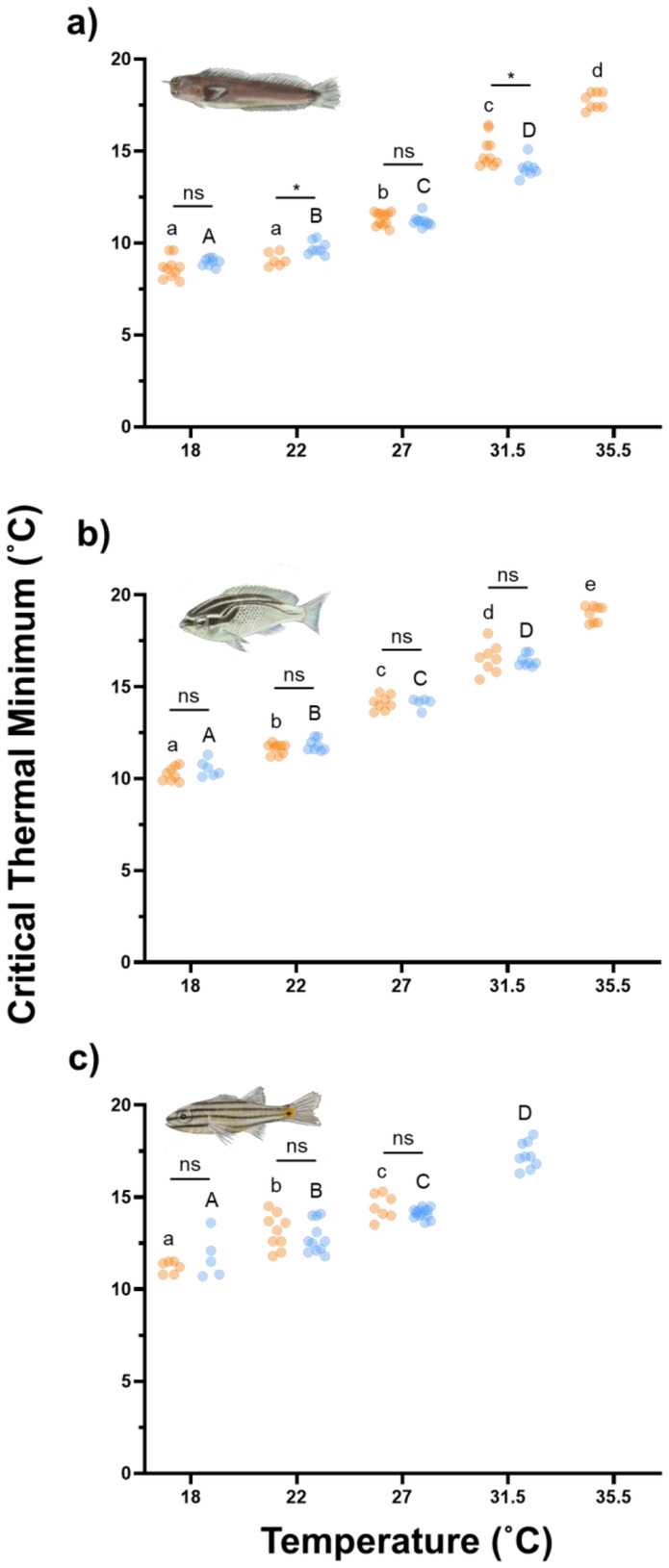
Critical thermal minimum of *Ecsenius pulcher* (a), *Scolopsis ghanam* (b) and *Cheilodipterus novemstriatus* (c) from the Arabian Gulf (orange) and the Gulf of Oman (blue) at five temperatures. Lowercase letters denote significant differences (fdr < 0.05) between temperature treatment groups in fishes from the Arabian Gulf. Uppercase letters denote significant differences (fdr < 0.05) between temperature treatment groups in fishes from the Gulf of Oman. Asterisks denote significant differences (*fdr < 0.05) between fishes from the different regions at a given temperature. *N* = 79, 68 and 61 for *E. pulcher* , *S. ghanam* and *C. novemstriatus* . Where data are present, *n* = 5–14 per species, per region and per temperature.

Contrary to our prediction, CT_min_ at the coldest acclimation temperature (18.0°C) did not differ between fishes from the AG and the GO (Figure 4, *E. pulcher* CT_min_ ± SE, AG = 8.65 ± 0.19, GO = 8.96 ± 0.075, *p* = 0.14; *S. ghanam* CT_min_ ± SE, AG = 10.25 ± 0.14, GO = 10.55 ± 0.18, *p* = 0.40; *C. novemstriatus* CT_min_ ± SE, AG = 11.20 ± 0.13, GO = 11.74 ± 0.53, *p* = 0.30).

**FIGURE 5:**
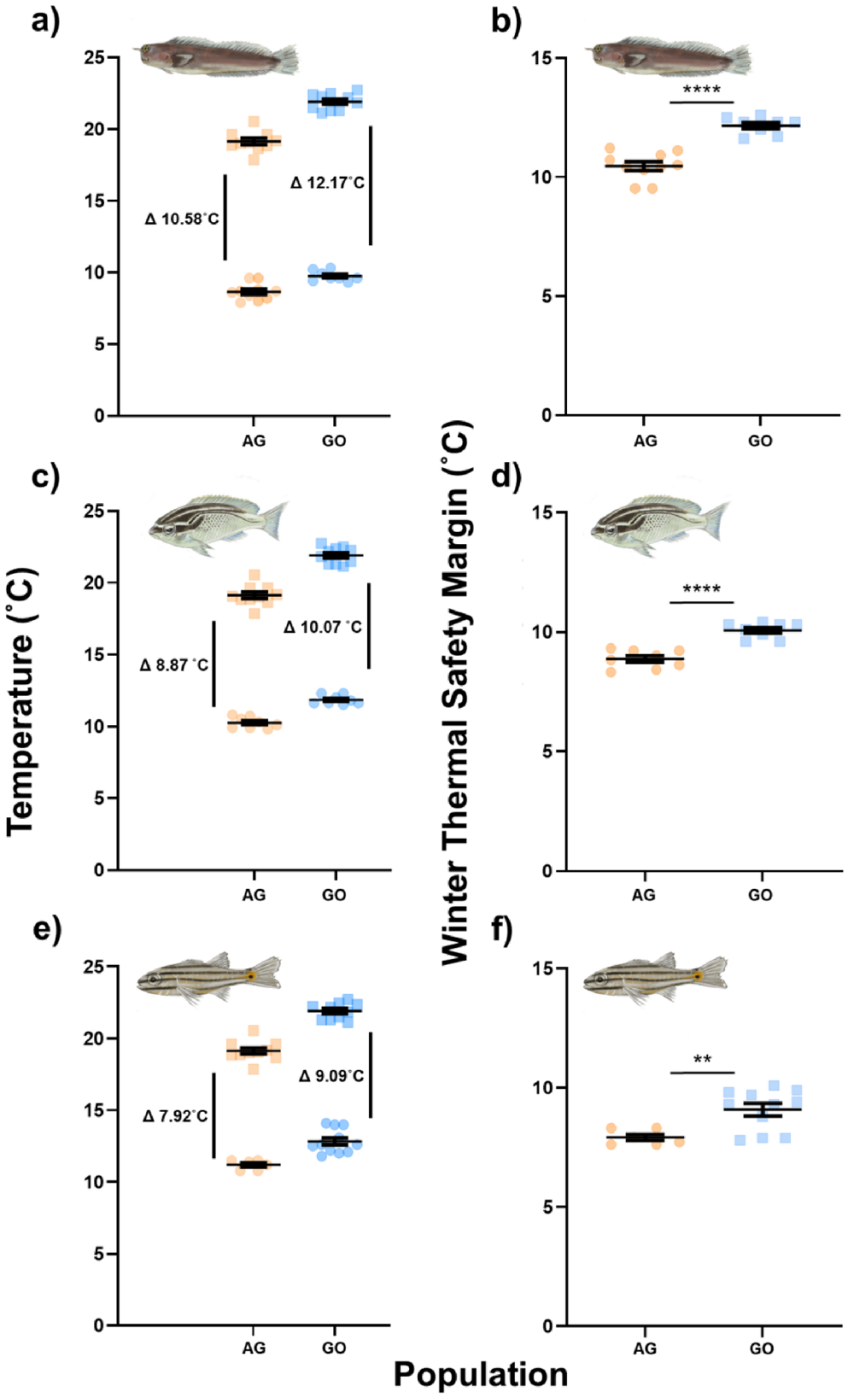
(a, c, e) Critical thermal minimum (circles) and minimum annual sea surface temperatures (squares, 2010–2019). (b, d, f) Winter thermal safety margins. Data show *Ecsenius pulcher* (a, b), *Scolopsis ghanam* (c, d) and *Cheilodipterus novemstriatus* (e, f) from the Arabian Gulf (orange) and the Gulf of Oman (blue) naturally acclimatised to their respective winter temperatures (Arabian Gulf, 18.0°C; Gulf of Oman, 22.0°C). Delta represents the mean thermal safety margin (environmental minimum temperature—CT_min_). *n* values: *E. pulcher* , AG = 10, GO = 8; *S. ghanam* , AG = 8, GO = 8; *C. novemstriatus* , AG = 6, GO = 11. Bars and error bars show the mean ± standard error.

Similar to summer conditions, all three species of AG fishes displayed significantly lower TSMs than conspecifics from the GO at their respective winter acclimation temperatures (AG = 18.0°C, GO = 22.0°C, Figure 5, *E. pulcher* TSM ± SE, AG = 10.58 ± 0.17, GO = 12.17 ± 0.13, *t* = 7.32, df = 15, *p* < 0.0001; *S. ghanam* TSM ± SE, AG = 8.87 ± 0.14, GO = 10.07 ± 0.11, *t* = 6.71, df = 14, *p* < 0.0001; *C. novemstriatus* TSM ± SE, AG = 7.92 ± 0.13, GO = 9.09 ± 0.26, *t* = 3.17, df = 15, *p* = 0.0063). On average, TSMs during the winter were 1.35°C lower in AG fishes compared to those from the GO. Across all species in the AG, winter TSMs were markedly greater than summer TSMs (average winter TSM = 9.09°C, average summer TSM = 3.56°C).

### 3.2 Metabolic Rates

**FIGURE 6:**
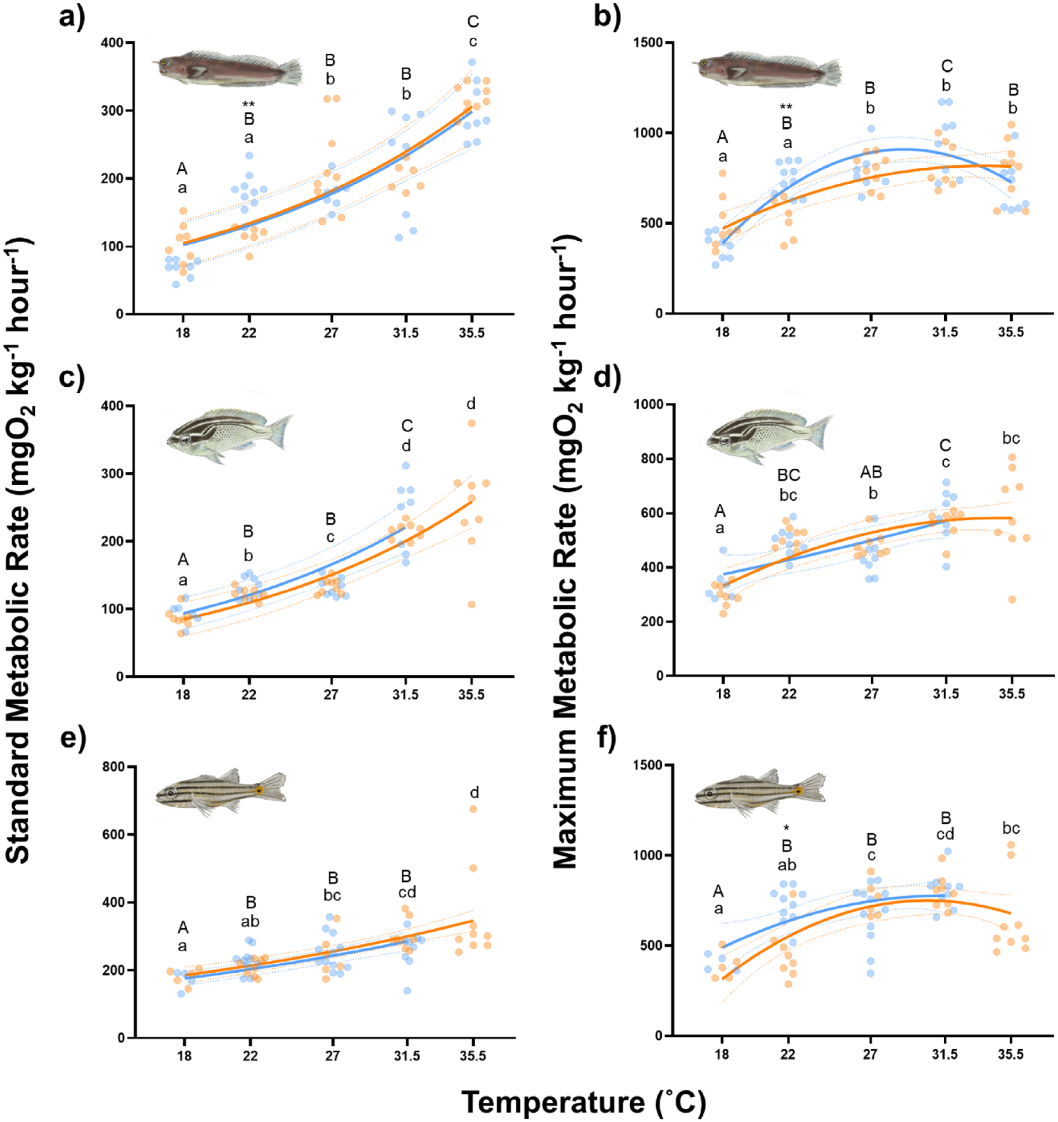
Mass‐scaled standard and maximum metabolic rates of *Ecsenius pulcher* (a, b), *Scolopsis ghanam* (c, d) and *Cheilodipterus novemstriatus* (e, f) from the Arabian Gulf (orange) and the Gulf of Oman (blue) at five temperatures. Curves for standard and maximum metabolic rates were fitted using exponential (SMR, *r* ^2^ range 0.83–0.99) and second‐order polynomial (MMR, *r* ^2^ range 0.31–0.64) equations, respectively, and the 95% confidence intervals are represented by the dotted lines. Lowercase letters denote significant differences (fdr < 0.05) between temperature treatment groups in fishes from the Arabian Gulf. Uppercase letters denote significant differences (fdr < 0.05) between temperature treatment groups in fishes from the Gulf of Oman. Asterisks denote significant differences (*fdr < 0.05) between fishes from the different regions at a given temperature. *N* = 80, 71 and 71 for *E. pulcher* , *S. ghanam* and *C. novemstriatus*, respectively. Where data are present, *n* = 4–12 per species, per region and per temperature.

In accordance with our prediction, MMR showed no regional‐level differences at any temperature in *S. ghanam* (Figure 6d), and very few differences in *E. pulcher* and *C. novemstriatus* , with the only significant changes occurring at 22°C (Figure 6b,f, *E. pulcher*, MMR ± SE at 22°C, AG = 518.9 ± 45.41, GO = 747.5 ± 27.48, *p* = 0.0022; *C. novemstriatus* , AG = 458.0 ± 54.47, GO = 724.1 ± 32.14, *p* = 0.012). Region displayed a significant effect on the overall model for MMR in *E. pulcher* (*p* = 0.043, *t*‐value = −2.07) and *C. novemstriatus* (*p* = 0.040, F‐statistic = 4.17), but not *S. ghanam* (*p* = 0.96, F‐statistic = 0.021). The Q10 values for MMR across the commonly experienced temperature range (22.0°C–31.5°C) were comparable between the AG and GO regions ( *E. pulcher* , AG = 1.63, GO = 1.29; *S. ghanam* , AG = 1.11, GO = 1.21, *C. novemstriatus* , AG = 1.80, GO = 1.12). Thus, while the MMR data largely follow predictions made through the tenet of ‘concrete ceilings’, some minor regional effects, specifically at the colder end of the TPCs, are present.

**FIGURE 7:**
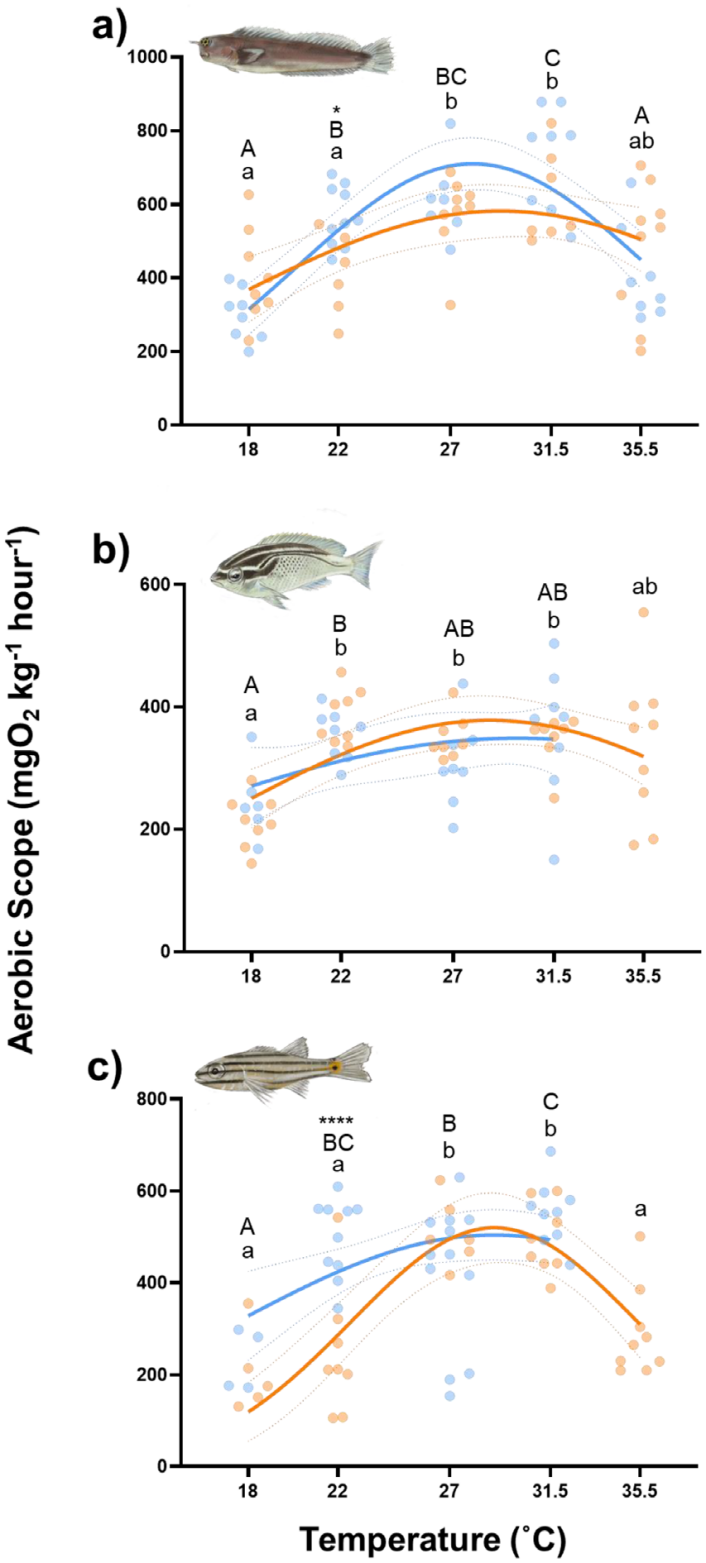
Mass‐scaled aerobic scope of *Ecsenius pulcher* (a), *Scolopsis ghanam* (b) and *Cheilodipterus novemstriatus* (c) from the Arabian Gulf (orange) and the Gulf of Oman (blue) at five temperatures. Curves were fitted using a Gaussian function (AS, *r* ^2^ range 0.13–0.58), and the 95% confidence intervals are represented by the dotted lines (Gaussian fit, mean and SD: *E. pulcher* , AG mean = 29.2, SD = 11.8, GO mean = 28.0, SD = 7.8; *S. ghanam* , AG mean = 28.7, SD = 11.7, GO mean = 29.9, SD = 16.7, *C. novemstriatus* , AG mean = 28.9, SD = 6.4, GO mean = 36.2, SD = 18.7). Lowercase letters denote significant differences (fdr < 0.05) between temperature treatment groups in fishes from the Arabian Gulf. Uppercase letters denote significant differences (fdr < 0.05) between temperature treatment groups in fishes from the Gulf of Oman. Asterisks denote significant differences (*fdr < 0.05, ****fdr < 0.0001) between fishes from the different regions at a given temperature. *N* = 80, 71 and 71 for *E. pulcher* , *S. ghanam* and *C. novemstriatus*, respectively. Where data are present, *n* = 4–12 per species, per region.

However, contrary to our predictions, both *S. ghanam* and *C. novemstriatus* showed no regional differences in SMR across the temperature range common to both regions (Figure 6c,e, 22°C–31.5°C), whereas *E. pulcher* showed differences at 22°C only (Figure 6a, *E. pulcher*, AG = 114.8 ± 6.32, GO = 179.5 ± 8.99, *p* = 0.0055). The overall models showed no significant effect of region on SMR in *E. pulcher* (*p* = 0.098, *t*‐value = −1.68), *S. ghanam* (*p* = 0.28, F‐statistic = 1.16), or *C. novemstriatus* (*p* = 0.54, *t*‐value = −0.61). The Q10 values for SMR across the commonly experienced temperature range (22.0°C–31.5°C) were comparable between the AG and GO regions ( *E. pulcher* , AG = 1.88, GO = 1.24; *S. ghanam* , AG = 1.82, GO = 1.86, *C. novemstriatus* , AG = 1.48, GO = 1.16). Concomitantly, there were also no regional differences in the AS of *S. ghanam* across the commonly experienced temperature range (Figure 7b), whereas *E. pulcher* and *C. novemstriatus* showed differences in AS at 22°C only, matching the MMR data (Figure 7a,c *E. pulcher*, AG = 408.5 ± 46.08, GO = 566.9 ± 25.61, *p* = 0.028; *C. novemstriatus* , AG = 246.4 ± 49.52, GO = 498.0 ± 27.08, *p* < 0.0001). Thus, the SMR data contradict our prediction formulated within the ‘plastic floors and concrete ceilings’ framework, that reef fishes in the AG would show a lower SMR at a given acclimation temperature than conspecifics from the GO and that the thermal sensitivity of SMR would differ between regions.

## 4 Discussion

Physiological plasticity is rapidly becoming a key aspect of climate change survival in coral reef ecosystems that have evolved under thermally stable conditions. Leading hypotheses, developed in eurythermal fishes under increasing average temperatures, suggest that basal physiological parameters show considerable thermal plasticity over long‐term timeframes, while physiological maxima remain relatively fixed (Sandblom et al. 2016). However, this is yet to be tested in tropical systems experiencing growing thermal variability under climate change. Utilising the world's hottest and most thermally variable coral reefs (AG) as a natural laboratory, where annual temperatures range from ~18.0 to 36.5°C, we assess the utility of the ‘plastic floors and concrete ceilings’ hypothesis for predicting the response of reef fishes to life in a thermally variable habitat across multiple generations. Our data reveal that three species of tropical reef fishes, with distinct ecological roles and separated by ~112 million years of evolution (Kumar et al. 2022), show conserved physiological responses that do not adhere to the principle of ‘plastic floors and concrete ceilings’. Specifically, we found minimal differences in baseline metabolic demands in any of the three species across their commonly experienced temperature range. Additionally, compared to fishes from typical present‐day thermal conditions for tropical coral reefs globally (e.g., the GO), those from the AG showed enhanced upper thermal limits (Figure 2), and 2 of 3 species showed a broader right‐hand side of the TPCs for AS, which may facilitate their survival on the thermally variable and extreme AG reefs. Importantly, this lack of regional differences in basal metabolic performance contradicts predictions made within the ‘plastic floors and concrete ceilings’ framework, suggesting that its application may not extend to tropical species experiencing changes in thermal variability.

In line with our predictions, fishes from the AG displayed enhanced upper thermal limits compared to conspecifics from the GO. These enhanced upper thermal limits manifest as either a higher CT_max_ (Figure 2) and/or an ability to acclimate to warmer (35.5°C) temperatures. The greater upper thermal limits in the AG fishes may play an important role in facilitating survival throughout the summer, where bottom‐water temperatures exceeding 36.0°C have been recorded on multiple reefs (Burt et al. 2019; Burt and Paparella 2023). Despite the differences in upper thermal limits, no regional effects on cold tolerance were observed at the coldest acclimation temperature (18.0°C) across any of the species. This asymmetry in the response of upper and lower thermal limits suggests that peak summer temperatures may be having a greater effect than winter lows. Indeed, the average summer thermal safety margin in AG fishes (3.54°C) is under half that of the average winter thermal safety margin (9.11°C), suggesting the pressures affecting these two traits are disparate.

Despite their greater upper thermal limits, fishes from the AG have lower summer TSMs than those in the GO (Figure 3) as the regional differences in environmental thermal variability exceed the differences in acute thermal limits. This suggests that AG fishes are more susceptible to further thermal variability (e.g., due to heatwaves) and that, while upper thermal limits are somewhat plastic in reef fishes, their rates of change may not be sufficient to totally mitigate projected long‐term increases in thermal variability. This erosion of summer TSMs is of particular concern in the AG, where long‐term warming is occurring above the global average rate and marine heatwaves are both frequent and severe (Burt 2024; Lachkar et al. 2021).

Previous studies have shown similar population‐level differences in upper thermal limits (Fangue et al. 2006; Chen et al. 2018; Sandblom et al. 2016). For example, redband trout ( *Oncorhynchus mykiss gairdneri* ) from desert populations show a higher CT_max_ than conspecifics from the cooler montane rivers, even when reared under identical conditions (Chen et al. 2018). Thus, changes in CT_max_ are a common response to the divergence of thermal environments over multiple generations, and these changes are potentially driven by adaptive evolution. Tropical fishes are thought to have low adaptive potential, with previous studies demonstrating changes of 0.04°C per generation for CT_max_ (Morgan et al. 2020). At this rate of change, a 3°C increase in maximum environmental temperature would require 75 generations of adaptation, a timeframe likely not afforded to most fishes under the current pace of climate change. Despite this, we show that fishes from the world's hottest coral reefs, after at least 6000 years of potential acclimation and/or adaptation, do display enhanced upper thermal limits compared to conspecifics from a nearby reference region.

Previous studies on the long‐term effects of warming have shown that MMRs are relatively fixed at so‐called ‘concrete ceilings’, but that basal physiological processes are more flexible and are often able to compensate for the acute effects of warming over multigenerational timeframes (Sandblom et al. 2016; Pilakouta et al. 2020; Jutfelt 2020). However, it is currently unclear whether populations experiencing long‐term changes in thermal variability also adhere to this framework. Comparing fishes in the AG to those in the GO, we see adherence to the principle of ‘concrete ceilings’, as MMR remains largely comparable across the two regions throughout the range of commonly experienced temperatures (22°C–31.5°C, Figure 6). However, contrary to expectations, we see few regional differences in SMR (Figure 6), despite long‐term differences in their experienced thermal regimes (Figure 1). These data suggest that tropical coral reef fishes experiencing increased thermal variability do not exhibit the ‘plastic floors’ commonly seen in response to long‐term elevations in average temperatures (Sandblom et al. 2016). Thus, while this hypothesis is useful in predicting the physiological response of temperate fishes to increasing average temperatures, its utility may not extend to tropical ecosystems experiencing changes in thermal variability too. Tropical coral reefs provide wide‐ranging ecosystem services including supporting fisheries, regulating nutrient cycling and sustaining the circa US$36 billion reef tourism industry (Woodhead et al. 2019; Spalding et al. 2017), and so it is crucial we develop suitable eco‐physiological frameworks to predict their response to ongoing climate change.

The lack of clear regional‐level plasticity in SMR has been observed in other species from the AG (Johansen et al. 2024) and could be explained by several factors. As well as the extreme summer highs (Figure 1b), the AG also experiences colder winters than the GO (Figure 1b), rendering it a more thermally variable environment (Figure 1d). Individuals with lower SMRs are thought to have a greater temperature sensitivity of metabolic rates (Norin and Metcalfe 2019; Norin et al. 2016), which may introduce additional pressures in the AG and preclude the shift toward reduced SMRs that is typically seen in chronically warmed populations (Sandblom et al. 2016; Pilakouta et al. 2020; Jutfelt 2020; Zambie et al. 2024). This highlights the importance of integrating predicted changes in thermal variability, as well as average temperatures, into studies of climate change. Alternatively, fishes on tropical coral reefs generally may be experiencing pressures that favour the reduction of SMR, masking any sign of changes unique to the AG fishes. If this is the case, it implies little flexibility in the rate at which SMR changes over time in populations experiencing different degrees of thermal extremes, which may restrict rates of adaptation. Finally, AG fishes may already be at the physiological limits for SMR acclimation. Understanding such constraints on the long‐term plasticity of SMR is crucial in determining the future of fishes in a warming world, where temperature‐induced restrictions of aerobic energy budgets are already touted as reducing body sizes (Johansen et al. 2024; Jutfelt et al. 2021; Queiros et al. 2024) and consequently population fecundity (Barneche et al. 2018; Hadj‐Hammou et al. 2024).

Despite regional differences in upper thermal limits, we found very limited evidence for the divergence of metabolic traits (SMR, MMR and AS) at any of the commonly experienced temperatures (Figures 6 and 7), suggesting that the two may not be casually linked. The acclimation of AG fishes to 35.5°C implies a broadening of the TPCs for metabolism compared to their conspecifics from the GO, where 2 of the 3 species were unable to acclimate to 35.5°C. Previous work has shown that the local adaptation of CT_max_ is sometimes accompanied by a broadening of the TPC for AS, allowing the maintenance of physiological O_2_ supply at greater temperatures (Chen et al. 2018). Other studies of local adaptation also demonstrate shifts in the TPC for AS. For example, the thermal optimum for AS in sockeye salmon ( *Oncorhynchus nerka* ) populations closely matches historical river temperatures (Eliason et al. 2011). Thus, changes in AS, manifesting as either a broadening or a right shift of the TPC, are a somewhat common response to multigenerational exposure to warming. Given that we observe no substantial regional differences across the commonly experienced temperature range, there is little evidence for a fully right‐shifted TPC, where increased performance at higher temperatures comes at the expense of reduced performance at lower temperatures (as previously suggested by Johansen et al. 2024). Rather, these data suggest that the ability of AG fishes to acclimate to 35.5°C, where two‐thirds of conspecifics from the GO cannot, represents a broadening of the right‐hand side of the TPC in some AG fishes. This change in the shape of the TPC may facilitate performance at higher temperatures, but without the accompanied loss of performance at the lower end of their thermal range. Thus, fishes in the AG appear to be more thermally generalist than their conspecifics from the GO, reflecting the greater seasonal variability they experience. If thermal limits are dictated solely by AS surplus during warming, then regional differences in thermal tolerance should be accompanied by a reshaping of the TPC for AS (Pörtner et al. 2017, but cf. Ern et al. 2016; Wang et al. 2014). However, *E. pulcher* showed enhanced upper thermal limits in the AG, but no change in the TPC for AS, adding to other studies in contradicting the universality of this assumed relationship (Ern et al. 2016; Wang et al. 2014). Instead, the data from *E. pulcher* agree with recent works in suggesting that the ultimate cause of organismal failure during acute heating is not always O_2_ limitation, but instead is due to species‐specific mechanisms (Ern et al. 2023).

Despite the tentative broadening of their TPCs for AS, *C. novemstriatus* from the AG displayed significant reductions in AS at 35.5°C compared to peak values (Figure 7), which may have critical ecological impacts. For example, a lower AS is thought to limit energy intake by reducing the metabolic capacity available for digestion (Auer et al. 2015; Jutfelt et al. 2021). Interestingly, changes in the dietary composition of fish occur in the AG during the summer (Shraim et al. 2017), alongside reductions in foraging time (D'Agostino et al. 2020), despite basal energetic demands increasing with temperature. Thus, there are signs that scope for feeding/digestion may already be limited at summer temperatures in some AG fishes (D'Agostino et al. 2020), an issue that could be driven by limitations in AS (Jutfelt et al. 2021). The potential broadening of the TPCs for AS may partially mitigate these declines, as it suggests some ability for coral reef fishes to rescue their AS through long‐term mechanisms (e.g., evolutionary, developmental, or transgenerational plasticity). However, this is unlikely to extend to total compensation given that *C. novemstriatus* from the AG shows a clear decline in AS throughout the summer temperatures, suggesting that they are living very close to their physiological upper limits.

The enhanced thermal tolerance conserved across AG fishes potentially demonstrates an adaptive response to thermal challenges that have persisted over millennia. However, while the three species examined here have managed to persist, both their winter and summer TSMs have shrunk and the overall diversity of fishes in the AG is substantially lower than that of the adjacent GO (Feary et al. 2010; Burt et al. 2011), suggesting that this plasticity may be far from ubiquitous. Furthermore, the recorded reductions in AS in some species at peak temperatures may have fitness consequences for fishes in the AG, further hindering their survival during this continued era of warming. This is especially pertinent as fishes in the AG that experience extreme seasonal temperature fluctuations appear to lack the ‘plastic floors’ typically relied upon to lower basal energetic demands during chronic warming (Sandblom et al. 2016; Pilakouta et al. 2020; Jutfelt 2020). Together, these data from the world's hottest and most thermally variable coral reefs provide valuable insight into the processes that may occur on coral reefs globally over the coming century, hinting that while some fishes possess the plasticity necessary to cope with warming over multigenerational timeframes, reductions in physiological performance and ultimately species richness, are likely in one of the most biodiverse ecosystems on earth.

## Author Contributions

**Grace O. Vaughan:** conceptualization, data curation, investigation, methodology, project administration, visualization, writing – original draft, writing – review and editing. **Daniel M. Ripley:** formal analysis, project administration, validation, visualization, writing – original draft, writing – review and editing. **Matthew D. Mitchell:** investigation, writing – review and editing. **Dain McParland:** methodology, writing – review and editing. **Jacob L. Johansen:** conceptualization, formal analysis, investigation, methodology, supervision, validation, writing – review and editing. **Holly A. Shiels:** conceptualization, supervision, writing – review and editing. **John A. Burt:** conceptualization, funding acquisition, resources, supervision, writing – review and editing.

## Conflicts of Interest

The authors declare no conflicts of interest.

## Data Availability

The data that support the findings of this study are openly available in Figshare at https://doi.org/10.6084/m9.figshare.28342562. The sea surface temperature data are available from the Physical Oceanography Data Archive at https://doi.org/10.5067/MODAM‐1D4N9.
